# SARS-CoV-2 and animals, a long story that doesn't have to end now: What we need to learn from the emergence of the Omicron variant

**DOI:** 10.3389/fvets.2022.1085613

**Published:** 2022-12-15

**Authors:** Alessandro Reggiani, Gianluca Rugna, Paolo Bonilauri

**Affiliations:** Istituto Zooprofilattico Sperimentale della Lombardia e dell'Emilia Romagna, Brescia, Italy

**Keywords:** SARS-CoV-2, wildlife, surveillance, diagnostic test, evolution, spillover, zoonosis

## Abstract

OIE, the world organization for animal health, recently released an update on the state of the art of knowledge regarding SARS-CoV-2 in animals. For farmed animals, ferrets and minks were found to be highly susceptible to the virus and develop symptomatic disease both in natural conditions and in experimental infections. Lagomorphs of the species *Oryctolagus cuniculus* are indicated as highly susceptible to the virus under experimental conditions, but show no symptoms of the disease and do not transmit the virus between conspecifics, unlike raccoon dogs (*Nyctereutes procyonoides*), which in addition to being highly susceptible to the virus under experimental conditions, can also transmit the virus between conspecifics. Among felines, the circulation of the virus has reached a level of cases such as sometimes suggests the experimental use of vaccines for human use or treatments with monoclonal antibodies. But even among wild animals, several species (White-tailed deer, Egyptian rousettes, and minks) have now been described as potential natural reservoirs of the virus. This proven circulation of SARS-CoV-2 among animals has not been accompanied by the development of an adequate surveillance system that allows following the evolution of the virus among its natural hosts. This will be all the more relevant as the surveillance system in humans inevitably drops and we move to surveillance by sentinels similar to the human flu virus. The lesson that we can draw from the emergence of Omicron and, more than likely, its animal origin must not be lost, and in this mini-review, we explain why.

## 1. Introduction

The *Coronaviridae* is a well-known and studied family of viruses in veterinary medicine, and there are several farmed, wild, or pet animal species that are infected or have diseases from this family of viruses. Bats are the wild animals that host the majority of known coronaviruses, including the virus associated with severe respiratory syndrome (SARS), named SARS-CoV-2 (1, 2). Having known this, when SARS-CoV-2 emerged as a pandemic virus of zoonotic origin, an intensive research for a possible intermediate host of the virus began, research that is still incomplete today (3–5).

We are entering the 4th year of the pandemic and although the SARS-CoV-2 has substantially been sustained by human-to-human transmission, there are many factors, not all of which have been clarified, that determine the extent of transmission of the virus in the human population. Human-to-human transmission is strongly increased when the emergence of highly transmissible variants occurs, the latest being Omicron. The Omicron variant (B.1.1.529), first identified in November 2021, quickly spread around the world, causing another global epidemic, but its origin remains unclear (6). Genetic studies on Omicron BA.1 and BA.2 protein S indicate a high affinity for the mouse ACE2 receptor, while the ancestral SARS-CoV-2 S-trimer binds better to the cat receptor than to the mouse receptor, suggesting a possible human-cat-mouse-human evolutionary path for the emergence of Omicron BA.1 and BA.2 (7, 8).

Nevertheless, it is certain that the SARS-CoV-2 virus is able to infect and transmit among many animal species. There are four documented cases of animal to human transmission: the first well-documented case was in mink, where human-to-mink, mink-to-mink and back mink-to-human transmission of SARS-CoV-2 was observed (9). Another case in which the evidence supports the animal to human transmission is that which took place in Hong Kong between hamsters and humans (10). Although the exact mechanism by which white-tailed deer were infected by SARS-CoV-2 has not yet been clarified, human-to-deer transmission is now well-established, such as deer-to-deer transmission and reverse transmission from deer to human is strongly suspected (11, 12). The latest case is supported by genetic studies and involved a Thai vet and a cat in 2021. The vet is suspected of having been infected after being sneezed on by a cat that belonged to positive owners (dad and son). The genetic variant involved was B.1.167.2, Delta (13).

Current knowledge indicates that wildlife does not play a significant role in the spread of SARS-CoV-2 in humans, but the circulation of the virus in wild animal populations can affect the health of these populations and can facilitate the emergence of new variants of the virus. Furthermore, when we shift our attention away from pets, from those kept in captivity, such as zoos, and from farmed animals, it is much more difficult to track and control infections in animals. This is especially the case for viruses circulating in wild animals, whose role in the current pandemic has not yet been clarified, and it will take a long time and much research to do so.

## 2. SARS-CoV-2 evolution

As soon as the first genomic sequence of SARS-CoV-2 became available, it was analyzed to determine its nature and its putative evolutionary path. Despite its origin has not been completely understood, it has been demonstrated that this virus descends from bat coronaviruses and could have been transmitted to humans via a still unknown intermediate host (14, 15). The main feature that has enabled SARS-CoV-2 to infect humans and spread around the world is its ability to bind the ACE2 receptor, which has been demonstrated to be an ancestral trait of sarbecoviruses, lost during the evolution of some clades, that is still evolvable and worth further attention (16). In contrast, the furin cleavage site in the spike protein, whose loss impairs viral pathogenicity (17), is a much rarer feature in other SARS-like viruses; its origin is still unclear, possibly obtained *via* recombination, and has raised debates about the natural origin of SARS-CoV-2 (18).

After a first wave of pandemic in early 2020, carried out by the original Wuhan strain, SARS-CoV-2 has begun to accumulate mutations, eventually leading to the generation of different linages, some of which have been declared as Variants Of Concern (VOC) by the WHO. Data from Nextstrain (https://nextstrain.org/ncov/gisaid/global/6m) show how these VOC have all evolved independently from the original strain, rather than one from another (19). The main source of mutations is the RNA-dependent RNA polymerase (RdRp) encoded in the viral genome, which can incorporate mutations into the genomic template during replication. In the context of a highly transmissible virus that recently jumped to humans, this has led to a growing adaptation to the new host *via* the selection of more fit clades. Moreover, as the time passed, more people became immune due to previous infections and/or vaccinations, thus adding a selective pressure to viral evolution. Finally, the role of recombination events between different linages of SARS-CoV-2 or with other related CoVs should be taken into account.

The first VOC (PANGO lineage B.1.1.7) was detected in November 2020 in the United Kingdom and later named Alpha by the WHO, showing a high number of mutations gained in a relatively short time, giving rise to the hypothesis of it being generated by the chronic, prolonged infection of immunocompromised hosts (20). The main of these mutations in the spike protein are the N501Y and a deletion of aminoacids 69 and 70. In particular, N501Y could be related to an adaptation of the virus to mice hosts (21). In any case, even though a second wave was due to Alpha, no significant reduction in vaccine efficacy was linked to this variant (22). In October 2020 a new VOC (PANGO lineage B.1.617.2) was identified in India, named Delta, whose increased fitness allowed it to rapidly replace Alpha and other locally circulating variants (like Gamma and Mu) to generate a third global pandemic wave in 2021. Delta bore new mutations that enhanced its transmissibility by 40–60% compared to Alpha (23) and enabled immune escape from therapeutical mAbs, convalescent sera, and vaccination (24).

In November 2021, a fifth VOC (PANGO lineage B.1.1.529) named Omicron was detected in South Africa, then rapidly spread around the world to give rise to a fourth pandemic wave between the end of 2021 and the beginning of 2022. Moreover, Omicron sublineages (identified, for nomenclature easiness reasons, with aliases such as BA.1 to BA.5) arose during 2022 until BA.5 became the most circulating variant globally in the past 6 months (https://nextstrain.org/ncov/gisaid/global/6m, accessed on 26/10/2022), also due to Omicron's shorter incubation periods and maybe milder clinical symptoms (25). Most of the sublineages known so far, whose description exceeds the purpose of this mini-review, are resumed and well-described in (26).

The main feature of the Omicron VOC is the higher number of mutations that it possesses, compared to the original Wuhan strain and to the previously circulating variants, initially harboring around 50 mutations, of which 32 are located in the spike protein (27). This rapid accumulation of mutations could be owed to two main different scenarios: first, the virus could have achieved a chronic infection in an immune-compromised patient, where it could generate variants without a potent cellular immune response and possibly under a positive selective pressure due to vaccination (28, 29); alternatively, SARS-CoV2 could have jumped back to some animal species and there, due to a different selective pressure, could have accumulated different mutations before getting to humans again. A mouse origin for Omicron has been proposed in a study that shows how pre-outbreak Omicron mutations are more consistent with an evolutionary history in mice rather than in human. This is also confirmed by an increased binding affinity with mouse ACE2 (30, 31). This adaptation could also be due to a different selective pressure exerted by some innate immunity intracellular effectors able to edit the viral genome, like APOBECs (32), ADAR (33), or ROS (34).

The hypothesis of a virus being able to infect and mutate in animal species has been confirmed in minks too. Su and colleagues (35) characterized some mutations in the spike protein of mink-derived viruses, such as Y453F and F486L, increasing the affinity for mink ACE2 while reducing that for human ACE2 (although maintaining infectivity). Interestingly, the circulation of these mink-adapted variants among humans dropped after mink culling policies were adopted (35). However, of all the mutations detected in mink-derived viruses in the Netherlands and in Denmark, 18 were found to be prevalent in both countries, and those were similar to human-derived mutations (36). Thus, due to its ability to cross the species barrier and propagate in animals, a One-Health approach should be implemented to identify putative SARS-CoV-2 reservoirs.

## 3. Surveillance in wildlife

The need for monitoring animal species to detect novel SARS-CoV-2 variants has been discussed in the literature as part of a One-Health approach (37). The likelihood of viral evolution and re-entry into the human population from wild animal reservoirs raises concerns about the infection of wildlife. Indeed, a SARS-CoV-2 sylvatic cycle, which may evade surveillance, could offer numerous chances for recurring spill-back into human populations and other susceptible wild species (38). Furthermore, because animals harbor several endemic CoVs (39), recombination events capable of introducing in SARS-CoV-2 genomic traits of increasing fitness could not be ruled out, as was the case with SARS-CoV and MERS-CoV (40), aside from being the hypothetical mechanism of SARS-CoV-2 emergence (41).

Our understanding of the virus, as well as its epidemiology and transmission, is still evolving. Natural and experimental infections in wildlife species may drive implementation of risk-based surveillance systems for SARS-CoV-2 in wildlife, by restricting the range of animal species to be monitored (42).

### 3.1. Natural infections in wildlife

**Figure 1:**
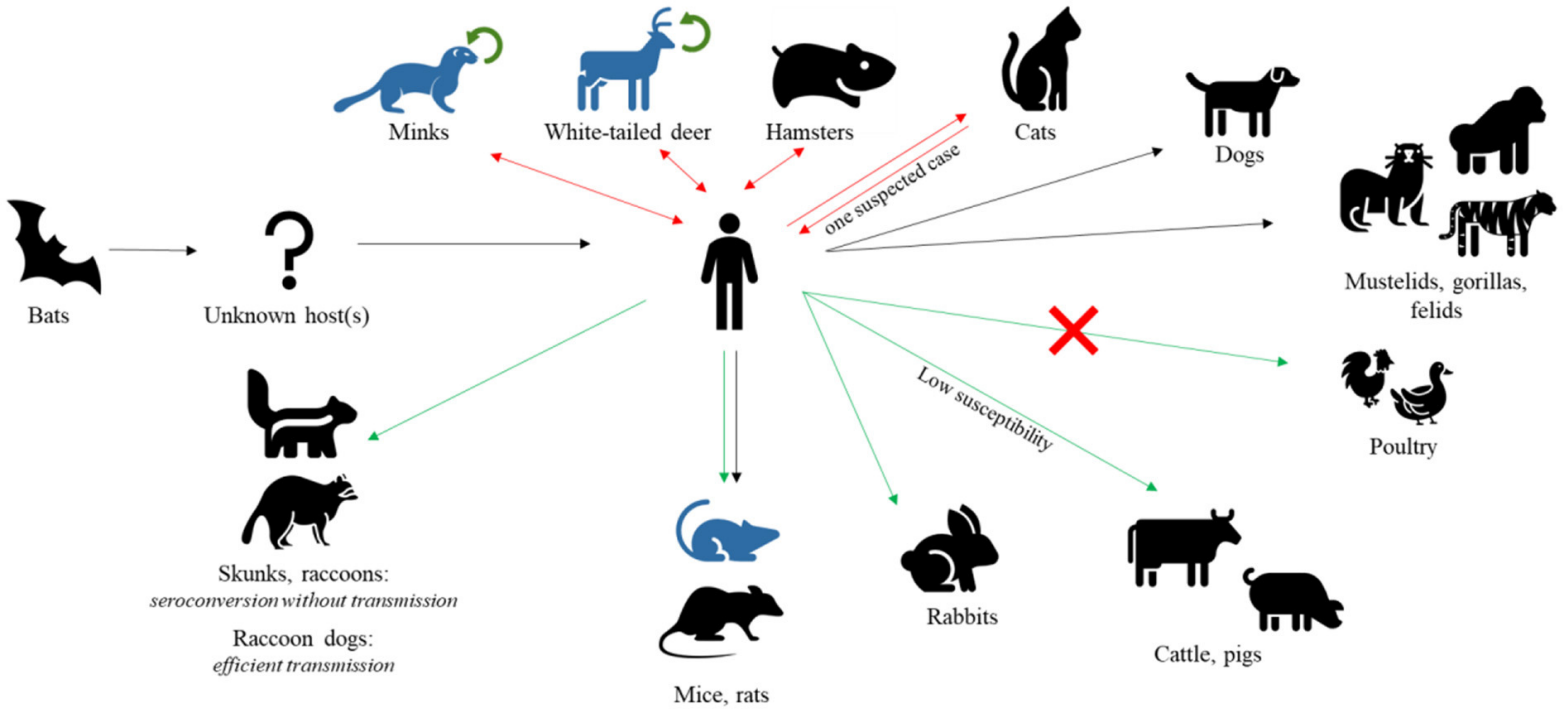
SARS-CoV-2 hosts. After jumping to human from bats, SARS-CoV-2 has been found in several other mammal species, naturally or experimentally infected. Black arrows represent natural infections, red double arrows represent natural infections with demonstrated reverse spillover to humans, green arrows represent experimental infections, and green circular arrows represent demonstrated intra-species circulation. Species with known adapted variants are indicated in blue. Notably, poultry are found to be insensitive to viral infection.

Although the contribution of wildlife to the emergence of SARS-CoV-2 is still unknown exactly (43), great concerns regarding the virus spilling-back and becoming endemic in animals have been expressed in light of several human-to-animal transmission events (Figure 1). To date, 735 SARS-CoV-2 animal events in 31 species were reported from 39 Countries in the SARS-ANI global dataset (44). SARS-CoV-2 has been demonstrated to have a wide host range, infecting a number of captive, farmed, and free-ranging wildlife species in addition to humans and domestic animals (45). Pigs and cattle have been found to be extremely insensitive to SARS-CoV-2 and poultry species are not at all susceptible to the virus (39). However, natural infections have been so far reported in a number of wild felids, in mustelids, e.g., Eurasian river otter (*Lutra lutra*), white-tailed deer (*Odocoileus virginianus*), and gorillas (46–52).

SARS-CoV-2 strains in wildlife generally mirrored the epidemiologic situation in humans; in early phase of pandemics animals were infected by ancestral strains, whereas in the latter stages diverse VOCs was detected, with Delta variant being the predominant (53).

In certain cases, human-to-animal transmission led to onward epizootic circulation of SARS-CoV-2, as in the case of farmed American minks reported from many European and American Countries (39, 54–56), and of white-tailed deer (*Odocoileus virginianus*) in North America (11, 12) (Figure 1). Transmission within animal populations may promote the origin of new variants as a consequence of evolutionary changes in the virus. Strain tracking by whole genome sequencing evidenced a mink-selected SARS-CoV-2 lineage, named Cluster 5, which has further spilled-back to humans (46, 57). Additionally, the transmission of a highly divergent SARS-CoV-2 strain (B.1.641) from a white-tailed deer to humans was recently suspected in Canada (58).

### 3.2. Experimental infections in wildlife

Since the beginning of the pandemics, researchers have been investigating the susceptibility of various animal species to SARS-CoV-2 (38). Many studies focused on peridomestic wild species, such as carnivores, mustelids, rodents, and chiropters, which live in close proximity to humans and are thus at increased risk of virus exposure (59).

Among peridomestic wildlife, the striped skunk (*Mephitis mephitis*) and the raccoon (*Procyon lotor*), were experimentally infected with the SARS-CoV-2 strain USA-WA1/2020; all inoculated animals seroconverted, suggesting that both species are susceptible to SARS-CoV-2 infection. However, it appeared unlikely for SARS-CoV-2 to establish itself in wild raccoon and skunk populations due to the lack of virus isolation from raccoons and the low amount of virus shed by skunks (38). Rabbits of the species *Oryctolagus cuniculus* have been also demonstrated susceptible to experimental infection with SARS-CoV-2, developing an asymptomatic infection with an acute phase of viral shedding. Even though there is no evidence of a natural infection in wild lagomorphs, these findings call for more research (60). In contrast, experimentally infected raccoon dogs (*Nyctereus procyonoides*) efficiently transmitted the virus to in-contact animals (61).

To date, two bat species, i.e., big brown bats (*Eptesicus fuscus*) and Egyptian fruit bats (*Rousettus aegyptiacus*), have been tested in the laboratory for virus susceptibility and shed capacity (62, 63). While one of the in-contact Egyptian fruit bats became infected after being exposed to inoculated animals, none of the experimentally infected big brown bats shed virus or transmitted it, nor did either species of bat develop clinical signs of disease. However, there are over 1,400 bat species worldwide and each species may respond differently to infection (64).

Research has shown a broad susceptibility of North American rodents to SARS-CoV-2 (65). Under experimental condition, deer mice of the species *Peromyscus maniculatus nebrascensis* and *P. m. rufinus*, could transmit the virus to naive conspecifics, implying that they could act as a wildlife reservoir for the virus (66). Rodent susceptibility to SARS-CoV-2 varies by species, which is not surprising given that rodents are the largest and most diverse order of mammals. Importantly, under experimental conditions, mice are not susceptible to early pandemic variants of SARS-CoV-2, but they are susceptible to certain VOCs such as B.1.1.7/Alpha, BA.1.1/Omicron, and especially B.1.351/Beta (67, 68). Recently, Omicron variant has been shown to infect minks, who can then effectively spread the virus to other conspecifics (69). Therefore, a continuous monitoring of newly emerged variants for their cross-species transmission is strongly recommended.

### 3.3. Diagnostic tests

Detecting viral RNA using a Real Time reverse transcription polymerase chain reaction (Real Time RT-PCR) test is currently the gold standard to detect an active SARS-CoV-2 infection (70). Several Real Time RT-PCR kits have been developed and approved, globally (71, 72). Reduction of false-negative as a result of SARS-CoV-2 rapid mutation, could be overcome by applying highly conserved region targets and multiple targets (73, 74). However, sensitivity of molecular tests is also influenced by pre-analytical factors, such as low viral loads, viral shedding time, and type of collected specimens. In addition, it should be considered the time of collection in passive surveillance, since sample degradation could occur between the death of the wild animal and sample collection.

Evaluating virus-specific antibodies is a rapid and affordable method to determine the status of the disease in individuals as well as carry out epidemiologic surveys in animal populations. The Spike (S)-protein and Nucleocapside (N)-protein are widely used in serological tests for SARS-CoV-2 infection. Due to the functional activity of the S-protein's receptor-binding domain (RBD), the level of anti-RBD antibody is generally regarded as a surrogate marker of the viral neutralizing potential of a serum. As a result, RBD-based serology assays are commonly used in human and veterinary clinical practice. Nevertheless, these tests are prone to decreased sensitivity because of the high rate of mutation for RBD epitopes, indicating the need of a timely updating based on the antigenic properties of the newly emerging SARS-CoV-2 variants (75). In contrast, N-based tests should be less affected because the N gene is the most conserved SARS-CoV-2 gene (76). However, comparison of two commercial N-based ELISAs and an RBD-based indirect ELISA, against virus neutralizing test (VNT), which is considered the gold standard for SARS-CoV-2 antibody detection, showed a lower sensitivity of both N-based assays (77).

Ideally, both serology and RT-PCR would be integrated into a surveillance program for SARS-CoV-2 in wildlife. Given the short duration of viral shedding, serologic investigations provide a more realistic picture of actual SARS-CoV-2 exposure in animals. However, detection of the virus allows for genome sequencing, which improves understanding of how SARS-CoV-2 evolves in animals (78, 79). Indeed, inter-species transmission and evolution in new hosts can also result in viral diversity, as seen during the human-mink-human transmission of SARS-CoV-2 (35) and the sustained transmission of the virus in the white-tailed deer populations (58).

## 4. Conclusion and proposals

Surveillance of zoonotic diseases in wildlife is challenging since it requires a lot of labor and is somewhat expensive, and there are often limited resources available. When adopting “One-Health” surveillance, the emergence of new diseases present opportunities for the development of novel surveillance techniques (80). To reduce costs or improve animal population coverage, it is crucial to evaluate novel sampling procedures, implement hazard-specific surveillance, and evaluate the efficacy of diagnostic tests, with the aim of avoiding loss of sensitivity even as new strains emerge, as it could come with SARS-CoV-2 (81–83).

A priority list of species groups for initial consideration can be created using the current evidence of natural and experimental infections. Additionally, the design of a wildlife surveillance system needs to be driven by a thorough understanding of animal demographics, social network structure, disease transmission paths, and a deep knowledge of the human-animal interface (84).

A large passive surveillance study on the circulation of the virus in wild animals is underway in northeastern Italy (Ministero della Salute, grant IZSLER PRC0062021), in which organs and fecal samples of numerous species will be analyzed. We expect to have enough coverage of animals and species analyzed by 2023 to provide the results to the scientific community.

However, we can already say that detecting the virus's circulation in these situations is particularly difficult, and as a result, efforts should be multiplied in various European regions and Countries in order to maximize the probability of detecting the virus's presence among wild animals and maximize our knowledge of a virus that will undoubtedly continue to circulate among human and animal populations indefinitely.

## Author contributions

All authors listed have made a substantial, direct, and intellectual contribution to the work and approved it for publication.
